# Co-application of biochar and nitrogen fertilizer reduced nitrogen losses from soil

**DOI:** 10.1371/journal.pone.0248100

**Published:** 2021-03-24

**Authors:** Xiuwen Li, Sutie Xu, Avishesh Neupane, Nourredine Abdoulmoumine, Jennifer M. DeBruyn, Forbes R. Walker, Sindhu Jagadamma

**Affiliations:** Department of Biosystems Engineering and Soil Science, University of Tennessee, Knoxville, Tennessee, United States of America; Tennessee State University, UNITED STATES

## Abstract

Combined application of biochar and nitrogen (N) fertilizer has the potential to reduce N losses from soil. However, the effectiveness of biochar amendment on N management can vary with biochar types with different physical and chemical properties. This study aimed to assess the effect of two types of hardwood biochar with different ash contents and cation exchange capacity (CEC) on soil N mineralization and nitrous oxide (N_2_O) production when applied alone and in combination with N fertilizer. Soil samples collected from a temperate pasture system were amended with two types of biochar (B1 and B2), urea, and urea plus biochar, and incubated for 60 days along with soil control (without biochar or urea addition). Soil nitrate N, ammonium N, ammonia-oxidizing bacteria *amo*A gene transcripts, and N_2_O production were measured during the experiment. Compared to control, addition of B1 (higher CEC and lower ash content) alone decreased nitrate N concentration by 21% to 45% during the incubation period while the addition of B2 (lower CEC and higher ash content) alone increased the nitrate N concentration during the first 10 days. Biochar B1 also reduced the abundance of *amo*A transcripts by 71% after 60 days. Compared to B1 + urea, B2 + urea resulted in a significantly greater initial increase in soil ammonium and nitrate N concentrations. However, B2 + urea had a significantly lower 60-day cumulative N_2_O emission compared to B1 + urea. Overall, when applied with urea, the biochar with higher CEC reduced ammonification and nitrification rates, while biochar with higher ash content reduced N N_2_O production. Our study demonstrated that biochar has the potential to enhance N retention in soil and reduce N_2_O emission when it is applied with urea, but the specific effects of the added biochar depend on its physical and chemical properties.

## Introduction

Farmers rely heavily on nitrogen (N) fertilizers to improve crop yield because N is one of the primary nutrients that plants need for growth and productivity. In 2016, more than 144 million tons of fertilizer N was applied to agricultural lands across the world [1]. But less than 50% of the applied N is taken up by crops [2] and the rest is lost, potentially contributing to eutrophication, lake acidification, biodiversity loss, and global warming [3]. Maintaining or improving crop productivity through fertilization while causing a minimal adverse impact on the environment is a global challenge.

If mineral N, ammonium-N (NH_4_^+^-N) and nitrate-N (NO_3_^-^-N), is not managed properly, significant N loss can occur through volatilization, denitrification, or leaching. Several strategies are proposed for efficient plant use of mineral N including selection of appropriate fertilizer types and right application rates, methods, and time; and the use of nitrification inhibitors (NI) [4, 5]. Although the use of NI may reduce N leaching and nitrous oxide (N_2_O) emission losses by slowing down the nitrification process [4], it can result in other environmental problems such as NH_3_ pollution [6, 7].

Biochar amendment in soil is considered an alternative strategy for improving N use efficiency [8, 9]. Biochar, the co-product produced from the thermal conversion of biomass to biofuel at high temperatures [10], is found to be effective in reducing N losses by physical and chemical sorption due to its higher specific surface area and charged surface functional groups [11]. The oxygenated carboxyl and carbonyl functional groups in biochar can reduce NH_4_^+^ availability by sorption, resulting in a decreased rate of nitrification. Additionally, hydroxyl and alkyl functional groups can control the availability of NO_3_^-^, resulting in reduced N leaching and N_2_O emission [12–15]. Other studies reported that biochar decreased soil mineral N concentration because it stimulated N immobilization and NH_3_ volatilization [16, 17] or facilitated the denitrification process by changing the microbial community structure [18, 19].

The inconsistent response of biochar to soil N transformation processes could be due to the differences in biochar properties, which is attributed, in part, to the differences in feedstock types and conversion conditions. The type of feedstocks used during the production of biochar significantly affects its ash content and C:N ratio [20]. For example, biochar produced from debarked wood chips generally has lower ash content and higher C:N ratio than biochar produced from poultry litter [17]. In addition to feedstocks, conditions used during biochar production play key roles in determining its physical and chemical properties. Conversion temperatures influence biochar pH, specific surface area, and surface functional groups [21]. For example, the pH, surface area, and percentage of aryl substituted functional groups of biochar increased when pyrolysis temperature increased from 200 to 700°C [12]. Both feedstock and temperature influence cation exchange capacity (CEC) due to the amount of negative charge from oxygen-containing acidic functional groups formed on the biochar surface [17]. Generally, more oxygen containing functional groups are expected in biochars produced from grasses because of the higher concentration of cellulose, alkaline salts, and alkaline metal oxides in grasses [22]. At temperatures higher than 600°C, the conversion of oxygen-containing functional groups to neutral or basic functional groups reduces CEC [23]. In general, biochar with a higher C:N ratio, higher CEC, and larger surface area reduces soil mineral N concentration because of the increased N immobilization and adsorption [17].

The effect of biochar amendment on N cycling also varies with geographic regions and ecosystems. For example, a lab incubation study by Thomazini et al. [24] reported that biochar prepared from hardwood increased NH_4_^+^ concentrations in agricultural soil from Florida but decreased NH_4_^+^ in forest soil from Minnesota. Thus, region and ecosystem specific studies are necessary to better understand the effect of biochar on soil N dynamics. In this study, we conducted a laboratory experiment using soils collected from a tall fescue-dominated pasture system in Middle Tennessee to determine how biochar influences soil N dynamics in the warm and humid southeastern US region. The specific objectives of this study were to determine the effect of two locally available biochar types with different physical and chemical properties—alone and in combination with urea—on (i) soil mineral N content and N_2_O production, and (ii) changes in microbial functional genes regulating nitrification process. We hypothesized that co-application of urea and biochar with higher CEC will decrease nitrification resulting in lower NO_3_^-^-N concentration and N_2_O production.

## Materials and methods

### Study site and soil sampling

Soil samples were collected from a tall fescue (*Festuca arundinacea*) dominated pasture in Lebanon, Tennessee, USA (36°11’45.3"N, 86°15’50.3"W) in December 2017. No special permissions were required to collect samples from this site. The mean annual temperature of this location is 14.5°C and the mean annual precipitation is 1342 mm [25]. The soil was silt loam in texture in the Bradyville series (fine, mixed, semiactive, thermic Typic Hapludalfs). Soil samples were randomly collected from 0 to 15 cm depth using a soil auger and composited. Fresh samples were sieved through a 2 mm sieve on the same day of collection and a sub-sample was used for the soil moisture determination using the gravimetric method. After storing another sub-sample at 4°C for the incubation experiment, the rest was air-dried to determine soil physico-chemical properties using standard laboratory protocols (Table 1). Briefly, soil carbon (C) and N concentrations were determined by the dry combustion method [26] using a CN analyzer (Elementar vario TOC cube, Langenselbold, Germany). Soil inorganic N (NH_4_^+^-N and NO_3_^-^-N) was measured using a Continuous Flow Analyzer (Skalar Analytical B.V., the Netherlands) after extracting 5 g soil with 25 mL 2M KCl solution [27]. Other important macro and micronutrients were extracted from soil using Mehlich-1 solution [28] and analyzed using Inductively Coupled Plasma-Optical Emission Spectroscopy (ICP-OES).

**Table 1:** Properties of the soil used for the incubation experiment.

Soil properties	Unit	Mean ± SE
**pH (H**_**2**_**O)**		6.30 ± 0.02
**Moisture content**	%	26 ± 0.23
**Total organic C**	g C kg^-1^	18.9 ± 2.57
**Total N**	g N kg^-1^	1.70 ± 0.06
**C: N ratio**		11:1 ± 0.08
**NH**_**4**_^**+**^**-N**	mg N kg^-1^	4.30 ± 0.02
**NO**_**3**_^**-**^-**N**	mg N kg^-1^	26.4 ± 0.61
**Mehlich I extractable P**	mg kg^-1^	1.84 ± 0.04
**Mehlich I extractable K**	mg kg^-1^	38.6 ± 0.27
**Mehlich I extractable Ca**	mg kg^-1^	970 ± 1.79
**Mehlich I extractable Na**	mg kg^-1^	9.10 ± 0.27
**Mehlich I extractable Mg**	mg kg^-1^	185 ± 4.48
**Mehlich I extractable Cu**	mg kg^-1^	1.31 ± 0.09
**Mehlich I extractable Zn**	mg kg^-1^	2.13 ± 0.07

### Biochar characterization

Two types of locally available biochar were used in this study. The first type was produced from mixed hardwood chips without bark in Lebanon, Tennessee by gasification at 700°C (hereafter called B1) and the second type was prepared from mixed hardwood chips with bark by Proton Power Inc. in Lenoir City, Tennessee by pyrolysis at 1100°C (hereafter called B2). The properties of both B1 and B2 are summarized in Table 2. Biochar pH was determined by a pH meter using 1:20 biochar:deionized H_2_O (w:v) [29]. Biochar moisture content was determined by ASTM International [30]. The surface area was determined based on CO_2_ adsorption using the Brunauer-Emmet-Teller (BET) theory [31]. Cation exchange capacity was determined according to [32] after slight modification, which included the use of 1 μm size filter paper and determination of K concentration by an Inductively Coupled Plasma-Optical Emission Spectroscopy (ICP-OES) (Spectro Ciros CCD). Total C and N concentrations were determined by the dry combustion method [26] using Elementar Vario TOC cube CN analyzer. Both types of biochar were sieved through a 4 mm sieve before being used for the incubation experiment.

**Table 2:** Physico-chemical properties of the two biochars.

Properties	Biochar 1 (B1)	Biochar 2 (B2)
**pH (H**_**2**_**O)**	10.4 ± 0.4	8.96 ± 0.3
**Total C (g C kg**^**-1**^**)**	830 ± 65	855 ± 58
**Total N (g N kg**^**-1**^**)**	10.5 ± 1.2	8.1 ± 1.0
**C:N ratio**	79:1 ± 1.4	105:1 ± 1.9
**Moisture content (%)**	54.1 ± 0.14	9.84 ± 0.08
**Surface area (m**^**2**^ **g**^**-1**^**)**	279 ± 0.60	295 ± 0.44
**Ash content (%)**	3.02 ± 0.02	7.31 ± 0.08
**Cation exchange capacity (cmol**_**c**_ **kg**^**-1**^**)**	202 ± 23.8	71.6 ± 9.25

### Microcosm experimental design

Fresh soils were pre-incubated at 25±1°C at its initial field moisture content (26%) in the dark for seven days before the experiment started. After the pre-incubation, 35 g soil was transferred into a specimen cup, which was placed into a 500 mL mason jar for incubation. Soil moisture was maintained at 26% throughout the 60-day incubation by adding Milli Q water with mini pipette every week, if needed, to their initial weight. There were four treatments with different biochar and urea addition ratios: (i) control (soil alone with no biochar or urea), (ii) urea to provide 150 mg N kg^-1^ soil, (iii) biochar plus urea (biochar to provide 75 mg N kg^-1^ soil + urea to provide 75 mg N kg^-1^ soil), and (iv) biochar to provide 150 mg N kg^-1^ soil. Treatment (iii) and (iv) had two sets each, for two different types of biochar, B1 and B2, thus giving six unique treatments. The amount of biochar and urea were selected based on an extensive literature review of similar experiments (e.g., [33, 34]). After mixing the biochar and/or urea with the soil using a glass rod, all the jars were tightly closed and incubated at 25±1°C in the dark. The jars were opened every four to six days and flushed with ambient air for 10 min using a small fan to maintain an aerobic environment. There were 72 jars in total with six treatments, four destructive sampling points, and three replications.

### Measurement of nitrous oxide production

Gas samples were collected 16 times from three replicate jars of each treatment on day 0, 1, 2, 4, 6, 8, 10, 13, 16, 21, 26, 31, 37, 43, 51, and 60. Samples were collected from the headspace through the sampling port on the center of the jar lids using a needle attached to a 20 mL polypropylene syringe. Headspace samples were stored in 12 mL pre-evacuated glass vials sealed with butyl rubber septa after flushing the vials with 10 mL samples. Air samples were also collected from the ambient atmosphere and stored in vials. The concentration of N_2_O in the samples was determined within a week of collection by a gas chromatograph (Model GC-2014, Shimadzu, Japan) with an electron capture detector. The amount of N_2_O production on day i was calculated as below:

$$N_{2}O_{dayi}=[{(N}_{2}O_{sample-dayi}-N_{2}O_{air-dayi})\times V]/m$$ (1)

where N_2_O_sample-dayi_ is the N_2_O concentration (mg N L^-1^) in the sample on day i, N_2_O_air-dayi_ is the N_2_O concentration (mg N L^-1^) in the atmosphere on day i, V is the headspace volume of the jars (L), and m is the dry mass of soils used for incubation (kg). Cumulative N_2_O emission was calculated by adding the N_2_O production from individual measurements.

### Destructive sampling and soil analysis

On days 0, 3, 10, 30, and 60, soils from three replicated jars were destructively sampled to measure soil mineral N and ammonia-oxidizing bacteria (AOB) *amo*A gene transcripts. Soil from each jar was divided into two subsamples, one of which was air-dried for mineral N analysis, and the other was frozen at -80°C for RNA extraction.

### RNA extraction and ammonia-oxidizing bacteria *amo*A gene quantification

The abundance of AOB *amo*A gene transcripts was determined on day 10 and day 60. The RNA from soil was extracted from 2 g frozen soil stored at -80°C using an RNeasy PowerSoil Total RNA Kit (Qiagen, Hilden, German) following the manufacturer’s protocol. Polymerase chain reaction (PCR) amplification of the RNA templates was performed in 20 μL reaction mixture consisted of 3 μL RNA template with primer pairs *amo*A-1F/*amo*A-2R to check for remaining DNA [35, 36]. The quality and quantity of extracted RNA was determined using Nanodrop One^C^ (Thermo Fisher, DE) to ensure high-quality RNA yield (nuclear acid concentration > 30 ng μL^-1^, A260/A230 > 1.7 and A260/280 > 1.8). SuperScript IV First-Strand Synthesis System (Thermo Fisher, MA) was used to synthesize cDNA with random hexamer primers (Invitrogen) according to the manufacturer’s protocol. After synthesis, cDNA was stored at -20°C.

Quantitative real-time PCR (qPCR) was carried out for quantifying the abundance of AOB *amo*A gene transcripts on a CFX Connect Real-Time PCR Detection System (Bio-Rad Laboratories Inc., Hercules, CA) using PowerUp SYBR Green Master Mix (Thermo Fisher, MA). A standard curve was generated from serial 10× dilutions of plasmid DNA from one representative clone containing the *amoA* gene. Triplicate analyses per sample were conducted in 20 μL reaction mixtures containing 10 μL of SYBR Green Master Mix, 0.5 μL of each primer, and 3 μL of cDNA template containing approximately 20–25 ng of cDNA. The *amo*A genes were quantified using the primer pairs *amo*A-1F/*amo*A-2R [35]. Negative controls were included in each run which used sterilized distilled water as the template instead of a cDNA sample. The qPCR conditions were as follows: 95°C for 3 min, 40 cycles of 60 s at 94°C, 45 s at 56°C, 60 s at 72°C, and 72°C for 10 min. The R^2^ values were 0.991–0.997, and the primer efficiencies were 71–72%.

### Calculations and statistical analysis

Net ammonification and net nitrification rates were calculated by Eqs (2) and (3), respectively [37, 38]:

$$\text{Net}\;\text{ammonification}\;\text{rate}\;(\text{NAR})=\left[{\text{c}({\text{NH}}_{4}{}^{+}-\text{N})}_{i+1}\text{-}{\text{c}({\text{NH}}_{4}{}^{+}-\text{N})}_{i}\right]/\Delta \text{t}$$ (2)

$$\text{Net}\;\text{nitrification}\;\text{rate}\;(\text{NNR})=\left[{\text{c}({\text{NO}}_{3}{}^{-}-\text{N})}_{i+1}\text{-}{\text{c}({\text{NO}}_{3}{}^{-}-\text{N})}_{i}\right]/\Delta \text{t}$$ (3)

where NAR and NNR are expressed as mg kg^-1^ day^-1^, respectively, *i* and *i*+1 are the initial and post-incubation time; c(NH_4_^+^-N)_*i*_ and c(NH_4_^+^-N)_*i*+1_ are the mean concentrations of NH_4_^+^-N on days *i* and *i*+1, respectively; c(NO_3_^-^-N)_*i*_ and c(NO_3_^-^-N)_*i*+1_ are the mean concentrations of NO_3_^-^-N on days *i* and *i*+1, respectively; Δt is incubation time (d) between day *i* and *i*+1.

Treatment effects on NH_4_^+^-N, NO_3_^-^-N, cumulative N_2_O emission, NAR, NNR and the gene copies were analyzed by the one-way analysis of variance (ANOVA) using GLIMMIX procedure in SAS (version 9.4 Cary, NC) with treatment as fixed effects and replication as random effects for each sampling point separately. The normality of the residuals was tested by Kolmogorov-Smirnov test and the homogeneity of variance was tested by Levene test at 5% significance level to ensure that the assumptions of ANOVA are met. Statistical differences among treatments were determined by Fisher’s protected least significant difference (LSD) at 5% significance level (*p* ≤ 0.05). Bivariate correlations between NH_4_^+^-N, NO_3_^-^-N, and AOB *amo*A gene transcripts were determined by Pearson’s correlation analysis. Before analysis, the abundance of *amo*A gene transcripts was logarithm transformed to attain approximate normal distribution.

## Results

### Soil ammonium nitrogen content

**Fig 1:**
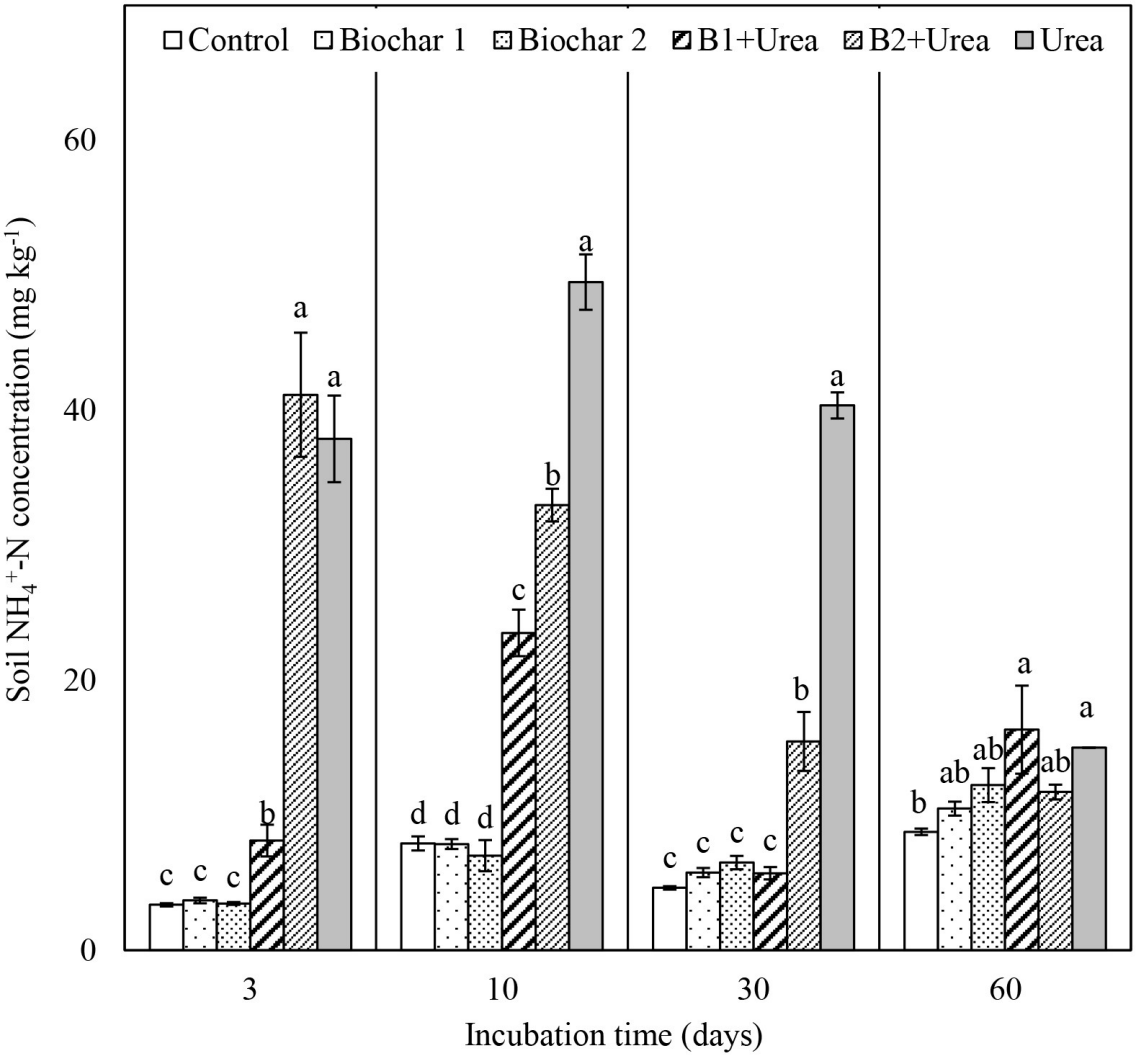
Changes in soil NH_4_^+^-N concentration from all the treatments on day 3, day 10, day 30 and day 60 of incubation. B1 and B2 refer to Biochar 1 and Biochar 2, respectively. Error bars represent standard error (n = 3). Different letters above the bars within each panel indicates a significant difference between treatments (*p* ≤ 0.05).

Urea alone significantly increased NH_4_^+^-N concentration in the soil during the first month of incubation (*p* < 0.05). The highest concentration of 49.5 mg N kg^-1^ soil was observed on day 10, followed by a decrease to 15 mg N kg^-1^ soil on day 60 (Fig 1). Co-application of biochar and urea resulted in a general reduction in NH_4_^+^-N content compared to the application of urea alone during the first 30 days, except in the case of B2 + urea on day 3. B1 + urea treatment resulted in 19.7%, 71.3% and 36.9% lower N concentration than B2 + urea treatment on day 3, 10, and 30, respectively. Although the total N contained in both biochar only treatments were similar to that in urea and biochar + urea treatments, soils amended with biochar only did not show any significant increase in NH_4_^+^-N concentration relative to control throughout the incubation period.

Between day 3 and 10, biochar alone treatments resulted in negligible NAR from -0.21 to 0.35 mg N kg^-1^ day^-1^ in B1 treatment and -0.28 to 0.27 mg N kg^-1^ day^-1^ in B2 treatment, which were significantly lower than that in all the urea added treatments during that period (1.27–12.3 mg N kg^-1^ day^-1^) (Table 3). Co-application of B2 and urea caused significantly higher NAR than B1 + urea treatment until day 30, but NAR was not significantly different between them on day 60. The highest NAR was observed in B2 + urea treatment on day 3 (12.3 mg N kg^-1^ day^-1^), which was ~10x higher than NAR from the B1 + urea treatment (1.27 mg N kg^-1^ day^-1^). However, by day 10 this difference in NAR between B1 + urea and B2 + urea was much smaller (2.87 vs. 2.22 mg N kg^-1^ day^-1^, respectively).

**Table 3:** Net Ammonification Rate (NAR) and Net Nitrification Rate (NNR) (mg N kg^-1^ day^-1^) for all treatments at different time point of the incubation.

Time	Control	B1	B2	B1+urea	B2+urea	Urea
**Net ammonification rate (mg N kg**^**-1**^ **day**^**-1**^**)**						
**Day 3**	-0.32 ± 0.02^c^	-0.21 ± 0.04^c^	-0.28 ± 0.02^c^	1.27 ± 0.23^b^	12.3 ± 0.89^a^	11.2 ± 0.62^a^
**Day 10**	0.36 ± 0.03^d^	0.35 ± 0.02^d^	0.27 ± 0.07^d^	2.22 ± 0.20^c^	2.87 ± 0.07^b^	4.52 ± 0.12^a^
**Day 30**	0.01 ± 0.00^d^	0.05 ± 0.01^c^	0.07 ± 0.01^c^	0.05 ± 0.01^c^	0.37 ± 0.04^b^	1.20 ± 0.02^a^
**Day 60**	0.07 ± 0.00^c^	0.10 ± 0.00^bc^	0.13 ± 0.01^ab^	0.20 ±0.03^a^	0.12 ± 0.01^ab^	0.18 ± 0.00^a^
**Net nitrification rate (mg N kg**^**-1**^ **day**^**-1**^**)**						
**Day 3**	-1.22 ± 0.13^c^	-2.84 ± 0.15^d^	1.07 ± 0.24^b^	0.28 ± 0.10^b^	3.33 ± 0.11^a^	3.94 ± 0.12^a^
**Day 10**	0.56 ± 0.08^e^	-0.26 ± 0.11^f^	1.56 ± 0.11^d^	3.16 ± 0.01^c^	5.13 ± 0.03^b^	5.63 ± 0.02^a^
**Day 30**	1.50 ± 0.06^cd^	0.43 ± 0.03^e^	1.24 ± 0.02^d^	1.71 ± 0.08^c^	4.68 ± 0.09^b^	5.94 ± 0.09^a^
**Day 60**	0.77 ± 0.03^d^	0.41 ± 0.05^d^	0.83 ± 0.03^d^	4.83 ± 0.05^b^	3.61 ± 0.04^c^	5.75 ± 0.16^a^

Treatments followed by different letters within each measurement time were significantly different (*p* ≤ 0.05).

### Soil nitrate nitrogen content

**Fig 2:**
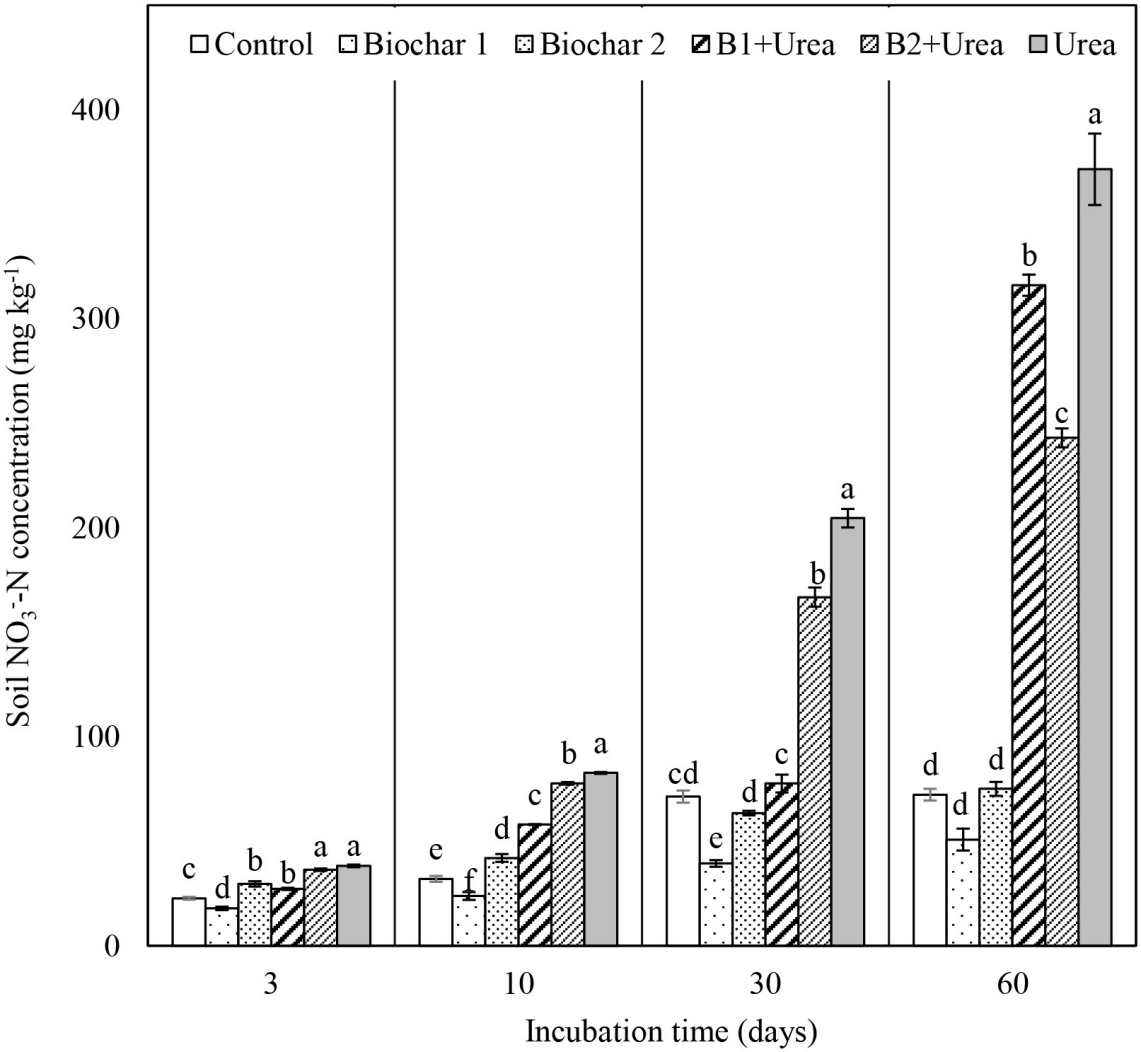
Changes in soil NO_3_^-^-N concentration from all treatments on day 3, day 10, day 30 and day 60 of incubation. B1 and B2 refer to Biochar 1 and Biochar 2, respectively. Error bars represent standard error (n = 3). Different letters above the bars within each panel indicates a significant difference between treatments (*p* ≤ 0.05).

The NO_3_^-^-N concentration in soil progressively increased in all treatments, including the control, as the length of incubation increased (Fig 2). All urea application treatments had significantly higher NO_3_^-^-N concentration in soil at day 60 compared to control and biochar only treatments. Urea alone application showed the highest increase in soil NO_3_^-^-N concentration from 38 mg N kg^-1^ on day 3 to 371 mg N kg^-1^ on day 60. Co-application of biochar and urea significantly reduced NO_3_^-^-N concentration compared to urea alone application in all time points except on day 3 when the concentration of NO_3_^-^-N in B2 + urea was not significantly different from urea alone treatment. When the two biochar treatments were compared, B1 + urea resulted in lower concentrations of NO_3_^-^-N than B2 + urea in the first 30 days. However, on day 60, B1 + urea had a 53% higher NO_3_^-^-N concentration than B2 + urea. Unlike the NH_4_^+^-N results, NO_3_^-^-N from B1 alone was lower than control during the first month while that from B2 alone was higher than control during the first 10 days. On day 60, B1, B2, and control had similar soil NO_3_^-^-N concentrations (Fig 2).

Soil amended with B1 alone had lower NNR than control during first 30 days while soil amended with B2 had higher NNR than control during first 10 days. When two types of biochar were compared, B1 amended soil had lower NNR than B2 during the first 30 days but their NNR was not significantly different at 60 days. Co-application of biochar and urea significantly reduced NNR compared to urea alone treatment at all measurements except during day 3 when NNR at B2 + urea co-application was not significantly different from urea alone treatment. B1 + urea had significantly lower NNR than B2 + urea in the first 30 days, while the NNR of B1 + urea was increased to the highest value (4.83 mg N kg^-1^ day^-1^) after 60 days, which is 33% higher than that of B2 + urea.

### Cumulative nitrous oxide emission

**Fig 3:**
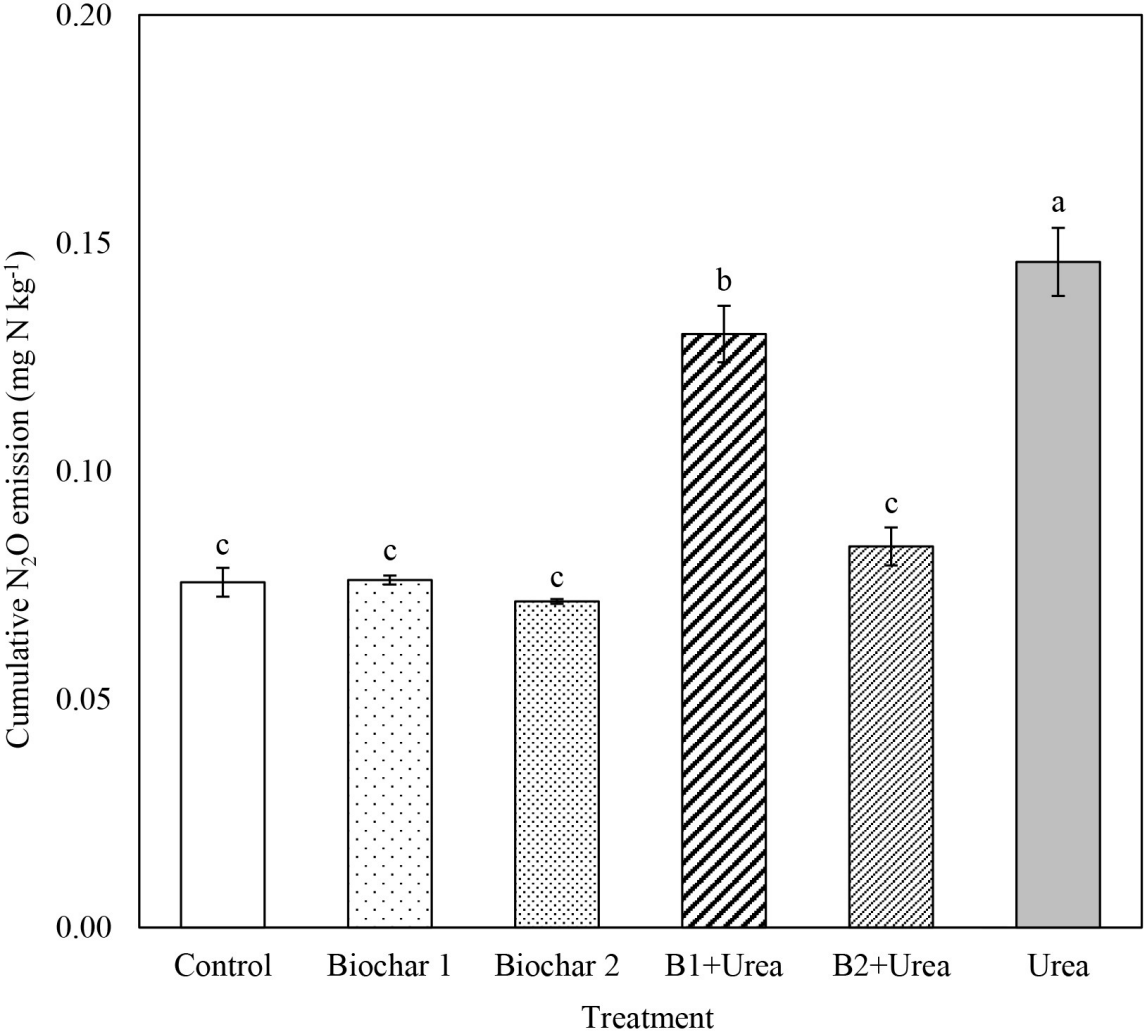
Cumulative N_2_O emission from all treatments at the end of incubation (day 60). B1 and B2 refer to Biochar 1 and Biochar 2, respectively. Error bars represent standard error (n = 3). Different letters above the bars within each panel indicates a significant difference between treatments (*p* ≤ 0.05).

Cumulative N_2_O emission after 60 days of incubation was significantly different among treatments (Fig 3). Urea application alone resulted in the highest amount of cumulative N_2_O emission (0.15 mg N kg^-1^ soil), which was 90% greater than the control. Co-application of B1 + urea (0.13 mg N kg^-1^) also resulted in 70% higher N_2_O emission than control. However, cumulative N_2_O production from B2 + urea, both types of biochar alone, and control did not differ significantly.

### The abundance of *amo*A gene transcripts

**Fig 4:**
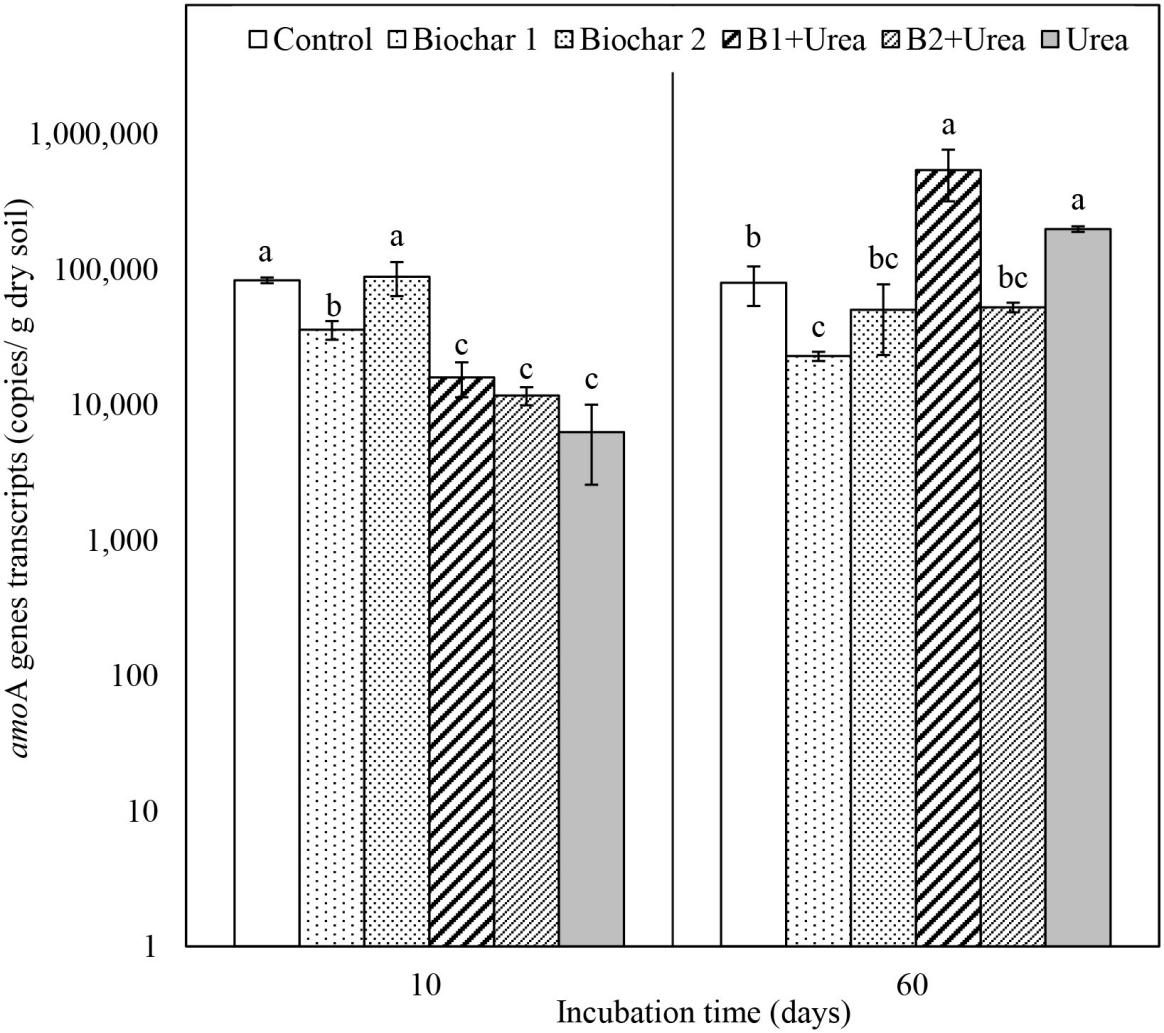
The abundance of AOB *amo*A gene transcripts from all treatments on day 10 and day 60 of incubation. B1 and B2 refer to Biochar 1 and Biochar 2, respectively. Error bars represent standard error (n = 3). Different letters above the bars within each panel indicates a significant difference between treatments (*p* ≤ 0.05).

**Fig 5:**
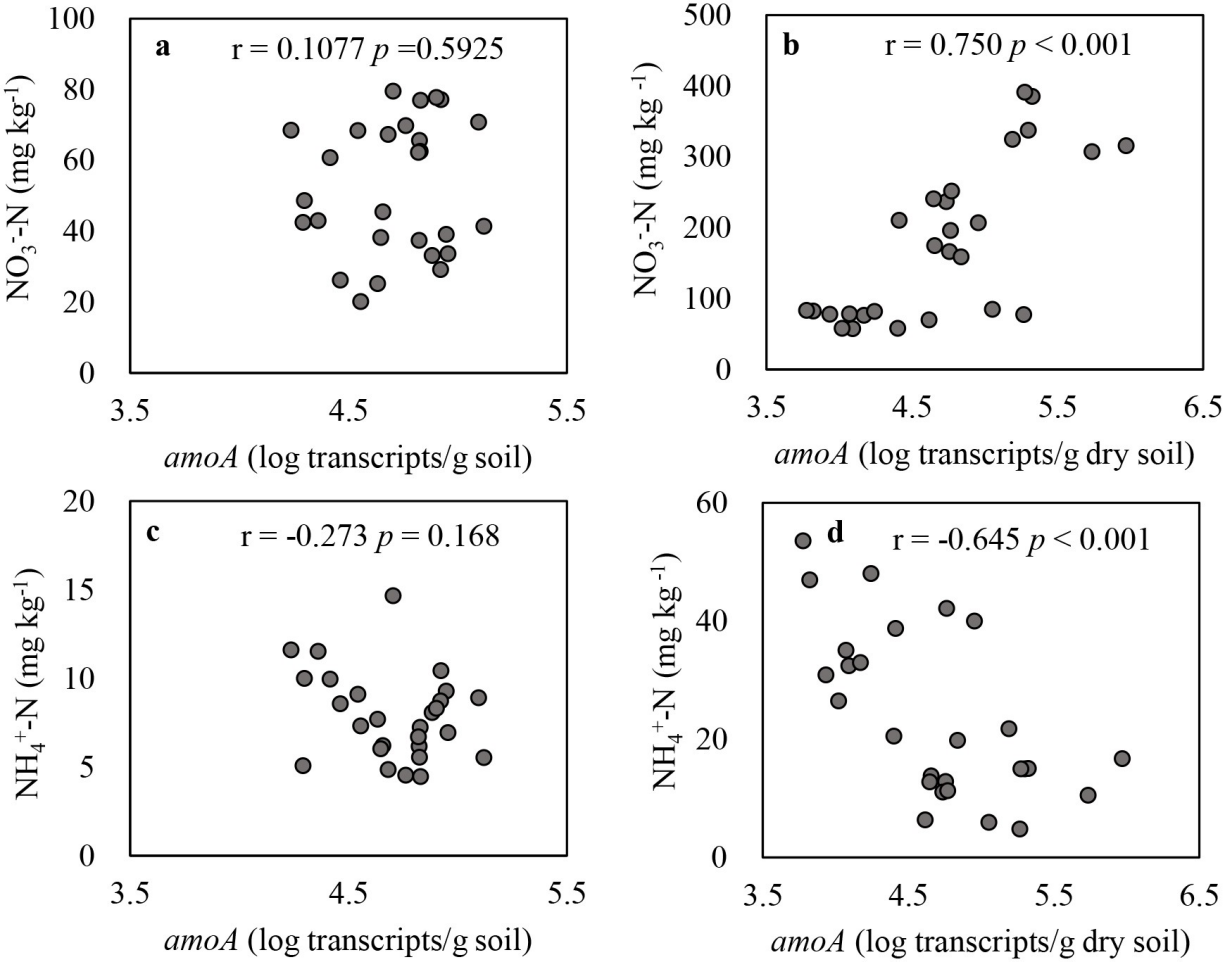
The correlation of *amo*A gene transcripts with NO_3_^-^-N and NH_4_^+^-N in treatments with no urea applied treatments; i.e. control and biochar alone (a, c) and with urea applied treatments; i.e. urea alone and urea-biochar co-application (b, d). r values are the result of Pearson correlation analysis.

The abundance of the AOB *amo*A gene transcripts in control ranged between 3.3×10^4^ and 8.3×10^4^ copies g^-1^ dry soil during incubation (Fig 4). Urea amendment, alone and in combination with biochar, significantly reduced the abundance of AOB *amoA* gene transcripts on day 10 compared to biochar alone and control treatments, but then increased at day 60, with values ranging from 6.3×10^3^ to 1.5×10^5^ copies g^-1^ dry soil. We also observed decreased abundances of transcripts in B1 alone treatment compared to control at both time points, which was not the case for the B2 treatment. After the 60-day incubation, the abundance of *amo*A transcripts in B1 treatment was 71% lower than control. The relationships between the logarithm transformed *amo*A gene transcript abundances and mineral N is shown in Fig 5. The *amo*A gene transcripts were positively and significantly correlated to NO_3_^-^-N concentration and negatively correlated to NH_4_^+^-N concentration in urea amended treatments (Fig 5b and 5d). No correlation was observed in control and biochar alone treatments (Fig 5a and 5c).

## Discussion

### Effect of urea and biochar on nitrogen mineralization

Soil NH_4_^+^-N concentration for all the urea treatments increased during the first 10 days (Fig 1) due to urea hydrolysis and then sharply decreased as nitrification continued (Fig 2), which is consistent with the results from other studies [39, 40]. The NAR was close to zero for the control and biochar only treatments but increased several-fold during the first 10 days for urea addition treatments, also indicating that soil NH_4_^+^-N concentration was increased mainly due to urea hydrolysis. Urea-added treatments resulted in higher substrate (NH_4_^+^-N) enhanced nitrification, evidenced by increased soil NO_3_^-^-N concentration (Fig 2), NNR (Table 3), and N_2_O emission (Fig 3). Urea addition can also influence nitrifier activity as ureolysis produces CO_2_, which can be a C source for nitrifiers to stimulate nitrification [41, 42]. However, our data showed that urea reduced the abundance of *amo*A gene transcripts that encode the enzyme which catalyzes the NH_4_^+^-N oxidation step in the nitrification process in the early phase of incubation (day 10) but increased their abundance after 60 days (Fig 4). The initial inhibition effect was probably caused by the excessive amount of NH_3_, which can be toxic to nitrifiers [43, 44]. This finding is consistent with the study by Staley et al. [45], which observed lower nitrifier diversity in the soil with a higher concentration of urea.

Compared to control, the addition of biochar B1 decreased nitrification (Fig 2, Table 3), which may have been due to the higher CEC of B1 and hence greater availability of exchange sites for NH_4_^+^ absorption [46]. No such decrease in nitrification was observed for B2, the biochar with relatively lower CEC. Previous studies also described a microbial mechanism for decreased nitrification by biochar application as biochar stimulated the activity of N-immobilizing heterotrophs, leading to the enhanced consumption of available NH_4_^+^ and overall inhibition of nitrification [47]. Wang et al. [48] found that biochar amendment slowed the nitrification process by reducing the abundance of AOB. In our experiment, the abundance of *amo*A gene transcripts was lower relative to control in the B1 treatment but not in B2 treatment (Fig 4), suggesting that the inhibition of nitrifier transcription by biochar depended on the type of biochar.

Biochar type also significantly influenced N transformation when biochar was co-applied with urea. For example, B1 resulted in lower NAR than B2 during the first 30 days, which could be attributed to the higher CEC of B1 compared to B2 (Table 1), leading to more urea absorption by B1. We also found a delay of NO_3_^-^-N production when B1 was co-applied with urea as it took a longer time to reach the maximum NNR (Table 3). This delay reiterates the ability of B1 to retain urea within their exchange sites longer than B2. A previous study reported that urea loading onto biochar surface resulted in slow and incomplete (70–80%) release of NH_4_^+^-N from urea [49]. Saha et al. [50] also reported that charcoal with higher CEC reduced urea mineralization. In addition, the slow release of urea can reduce NH_3_ toxicity, thus enhance long-term microbial activity [51]. This is supported by our finding that AOB *amo*A gene expression in B1 + urea was significantly higher than B2 + urea after 60 days (Fig 4).

Our results also showed that urea stimulated N mineralization by enhancing both ammonification and nitrification processes while biochar inhibited N mineralization by slowing nitrification. The mineral N (NH_4_^+^-N and NO_3_^-^-N) concentration in urea alone treatment after 60 days of incubation was 386 mg N kg^-1^, which was much higher than the sum of the added amount of N and the mineral N derived from the soil (150 mg N kg^-1^ from added N + 81 mg N kg^-1^ from soil, hereafter called expected mineral N concentration). This indicated that the urea application resulted in the increased transformation of organic N from soil to mineral N, exhibiting a positive priming effect. The enhanced organic N transformation was also observed when urea was co-applied with biochar. Compared to the expected mineral N concentration of 231 mg N kg^-1^, the amount of mineral N on day 60 in B1 + urea and B2 + urea treatments were 44% and 10% higher, respectively, despite only 75 mg N kg^-1^ was applied as urea. Similar to our findings, Baiga and Rao [39] and Fiorentino et al. [52] found increased soil mineral N concentration in biochar treatment, with or without urea, compared to the expected mineral N concentration. Some studies, however, showed evidence for no or negative effects of biochar on N mineralization when applied with N fertilizer [53].

### Effect of urea and biochar on nitrous oxides emission

As expected, urea treatment had the highest cumulative N_2_O emission after 60 days of incubation (Fig 3). Co-application of biochar and urea reduced N_2_O emission significantly compared to urea alone addition. However, the effect of biochar on N_2_O production was dependent on the biochar type. There was a significant reduction of N_2_O production per unit of synthetic N added for B2 but not for B1. Consequently, B1 + urea produced 89% of N_2_O as compared to urea alone despite the fact that only 50% of N was added as urea in B1 + urea treatment. Grutzmacher et al. [54] also reported that the application of biochar and N fertilizer together decreased N_2_O emission by 67–95% compared to N fertilizer alone treatment when the same amount of NH_4_^+^-N was added. The favorable effect of biochar in reducing N_2_O emission could be attributed to biochar’s ability to inhibit nitrification (see the previous section) and decreased N_2_O loss from denitrification processes. Some studies have attributed lower N_2_O production from biochar amendment to enhanced redox reaction that converts N_2_O to N_2_, which can be influenced by metal ions or organic radicals in the biochar [54–56]. This could be the potential dominant mechanism for the substantial reduction in N_2_O from the B2 + urea treatment in our study: B2 contains two times more ash content than B1 (Table 1) and thus possibly has higher metal ion content and ability to function as an electron shuttle [54]. Few other studies have reported that biochar reduces N_2_O production due to the entrapment of N_2_O on the biochar surface area, which slows down the gaseous diffusion [57].

### Abundance of *amo*A genes transcripts and its relationship with soil mineral N

Ammonia-oxidizing archaea (AOA) and AOB play active roles in nitrification as reported by several studies (e.g., [58–61]). In our study, we only focused on AOB because AOB outcompetes AOA under high N environments [61–64]. AOB contains genes encoding ammonia monooxygenase (AMO), one of the crucial enzymes responsible for the transformation of ammonia to nitrite. Among these genes, the putative catalytic polypeptide of AMO is encoded in *amo*A gene [65]. Thus, the abundance of *amo*A gene transcripts is an important indicator of the AOB activity in the nitrification process. The significant relationship between *amo*A gene transcripts and mineral N in urea added treatments (Fig 5b and 5d), but lack of correlation under the treatments without urea added (Fig 5a and 5c), indicates that AOB may have controlled N mineralization processes occurring in urea alone treatments due to the higher mineral N content from urea addition. The positive relationship between NO_3_^-^-N concentration and *amo*A gene transcripts (Fig 5b) clearly shows that more *amo*A genes were expressed as incubation progressed to convert ammonium from urea added treatments to nitrite. Other microbes, like ammonia-oxidizing archaea (AOA), may also have played an active role in nitrification as reported by several studies (e.g., [58–61]), but we only measured AOB. Although the relative importance of AOA and AOB in N mineralization process is unclear from our study, some other studies revealed that AOB outcompetes AOA under high N input while AOA is functionally dominant in soils with low N input [61–64].

## Conclusions

This study revealed that co-application of biochar and urea reduced both soil NH_4_^+^-N and NO_3_^-^-N concentrations and N_2_O production as compared to urea alone treatment, indicating that N mineralization rate can be reduced by adding synthetic N fertilizers together with biochar. When applied with urea, biochar with higher CEC reduced ammonification and nitrification rates, while biochar with higher ash content specifically reduced N_2_O production. Overall, applying synthetic N fertilizer and biochar together showed promise in reducing N losses from the system. Future studies are needed to understand the overall N balance in soils with biochar and urea additions. Studies are also needed to determine the effect of co-application of biochar and urea on N use efficiency in the field by taking into account the effect on plant growth, plant nutrient uptake, and nutrient losses.

## Supporting information

S1 Appendix Soil NO_3_^-^-N concentration, net nitrification rate, NH_4_^+^-N concentration, net ammonification, and abundance of amoA gene transcripts from all treatments during the incubation period. (XLSX)

S2 Appendix Cumulative N_2_O emission (mg/kg) from all treatments during the incubation period. (XLSX)
